# Dictionnaire des Termes de Médecine, Français—Anglais

**Published:** 1900-03

**Authors:** 


**Dictionnaire des Termes de Médecine, Français—Anglais.** Par H. de MÉRIC. Pp. vi., 243. London: Baillière, Tindall and Cox. 1899.

In the preface the author says that he has been reproached by some for including in the English-French predecessor of this volume many words not strictly belonging to medicine. So far from reproaching him for this laudable fulness, we think it a great recommendation. It is convenient for the medical reader to be able to find in the same dictionary terms belonging to the cognate sciences as well as those referring to his own technicality. Did we feel inclined to qualify our praise of this volume, it would be on the grounds that it is less full in explanation and less wide in comprehensiveness than its companion. At the end of the volume is a table of French weights and measures with their English equivalents, and some examples of prescriptions in both languages, which will prove useful to many, although the statement that "1 minim = 56 centimetre cubes" requires some alteration. We hail the completion of a work which distinctly fills up a gap in our medical dictionaries.

